# The Architects and the Medical Officers of Health

**Published:** 1901-08-17

**Authors:** 


### The Architects and the Medical Officers of Health.

If there is one elementary principle in sanitation on which we are all agreed it is as to the necessity for care in the planning, the execution, and the maintenance of all that concerns house drainage. Next in importance to good water come good drains. Hence the attention paid to these matters by the sanitary inspector, and hence not a little of the dislike so many people feel towards this often zealous but not always popular official. As a fact a very large proportion of the structural defects which are discovered by the sanitary inspectors of the health department are due to the erroneous planning or the imperfect laying of drains; in other words, they are defects which, with all respect to the architectural profession, we cannot but lay at the door of those by whom these drains have been planned, and by whom their construction is imagined to have been supervised. This being so, it is rather amusing to find that the Royal Institute of British Architects has been circularising the various metropolitan sanitary authorities suggesting that, in view of the recent creation of metropolitan borough councils, now is the time for removing from the health departments all matters relating to the construction, reconstruction, amendment and repairs of drains and sanitary appliances, and the structural removal of nuisances in connection therewith, and urging that all these matters shall henceforth be placed under the department of the surveyor to the authority, the "detection only" of nuisances arising from sanitary apparatus or drains remaining in the hands of the department of the medical officer of health.

Of course we quite enter into the architects' feelings on the subject. We must not forget, however, that, except for those non-æsthetic members of their profession who put up factories, workshops, and other buildings of utility, architects rather fancy themselves as artists—not as artists in drains, but as designers of things of beauty —and that, whatever advance the younger among them may have made in regard to sanitary affairs, drains are not, and probably never will be, regarded as the primary consideration in architectural designs. On every hand one cannot but see, even in regard to quite recently erected buildings, that matters of health are all too often made to give way to considerations of convenience, of expense, and of uniformity or picturesqueness of design, and we feel confident that if we want to have our drains right the plans should be passed by the medical department, whose sole object is that the building to be erected, however ugly it may be, shall at least be healthy.

If, moreover, we leave the question of what is right and turn attention merely to what is practically convenient, we see at once the evils which are inseparable from such a dual control over matters of drainage as is contemplated by the Royal Institute. Dr. Joseph Priestley, speaking recently to the Society of Medical Officers of Health on this subject said that where the drainage of new buildings is left with the surveyors' department and that of old ones with the public health department, as was done in years gone by and as still is done in some cases, difficulties arise from such dual control, and there is a chance of one department finding fault with another. To give an example he says: "A new building may be passed by a surveyor's department as fit for occupation, and, after a shorter or longer period of time, a case of infectious disease may occur there calling for the attention of a sanitary inspector, who may have to serve a notice in connection with a contravention or a non-enforcement of a bye-law, or on account of drainage being carried out in opposition to recognised present day ideas as to house-drainage construction." In this way owners are put to endless trouble and expense. We well remember a case in point. A medical officer, descanting on the difficulties under which he worked said: "There is a row of houses, they have only been put up a few years and I believe the drainage of every one of them is wrong. But I have no power to inspect until a case of fever arises. Then, of course, I have all the drains pulled out; but it will take a good many years and many cases of fever to put the whole row right. Yet all these plans were passed by the improvement committee." Such conflicts between the several departments of one authority are much to be deprecated. The obvious way out of the difficulty is that the entire control of the house drains should be placed in the hands of one department or the other; and if we are asked, Which? we can have no hesitation in saying that the department which will have to control them afterwards, namely the medical, should have the responsibility from the beginning.

If the suggestion of the Institute of British Architects were put into effect, the health department would have no say whatever in regard to the plans for the drainage of new buildings or with those drawn up for the amendment of the defects discovered by its own inspectors in old ones. Nothing could be done till all was finished, when, if the work did not come up to the standard, it would all have to be pulled out again. As Dr. Priestley says, "Sanitary inspectors must discover the nuisances, and the nuisances when discovered, must be abated to the satisfaction of the inspectors." Far better then let the sanitary department see to the whole thing at once, as is done in more than half the metropolitan boroughs at the present time.

